# Red Currant (*Ribes rubrum* L.) Fruit Waste Extract and Juice as Potential Spasmolytic Agents

**DOI:** 10.3390/plants14020234

**Published:** 2025-01-16

**Authors:** Maja Cvetković, Bojana Miladinović, Suzana Branković, Milica Randjelović, Slavoljub Živanović, Nemanja Kitić, Milica Milutinović, Miloš Jovanović, Dušica Stojanović, Haris Nikšić, Katarina Šavikin, Dušanka Kitić

**Affiliations:** 1 Department of Pharmacy, Faculty of Medicine, University of Niš, Dr. Zoran Djindjić Ave 81, 18000 Niš, Serbia; cvetkovicmaja987@gmail.com (M.C.); milica.randjelovic@medfak.ni.ac.rs (M.R.); milica.milutinovic@medfak.ni.ac.rs (M.M.); milos.jovanowic@gmail.com (M.J.); dusanka.kitic@medfak.ni.ac.rs (D.K.); 2 Department of Physiology, Faculty of Medicine, University of Niš, Dr. Zoran Djindjić Ave 81, 18000 Niš, Serbia; brankovic.suzana@yahoo.com; 3 Biomedical Research Centre, Faculty of Medicine, University of Niš, Dr. Zoran Djindjić Ave 81, 18000 Niš, Serbia; zivanovicyu@yahoo.com (S.Ž.); voidruner@gmail.com (N.K.); 4 Department of Social Medicine and Hygiene with Medical Ecology, Faculty of Medicine, University of Niš, Dr. Zoran Djindjić Ave 81, 18000 Niš, Serbia; dusica.stojanovic@medfak.ni.ac.rs; 5 Department of Pharmacognosy, Faculty of Pharmacy, University of Sarajevo, Zmaja od Bosne 8, 71000 Sarajevo, Bosnia and Herzegovina; harisniksic@gmail.com; 6 Institute for Medicinal Plants Research “Dr Josif Pančić”, Tadeuša Košćuška 1, 11000 Belgrade, Serbia; ksavikin@mocbilja.rs

**Keywords:** red currants, berries, juice, waste, anthocyanins, flavonols, antioxidative activity, spasmolytics, ileum, rats

## Abstract

Red currant (*Ribes rubrum* L.) berries are rich in bioactive compounds and exhibit nutritive and protective features. This research examined the content of secondary metabolites of the red currant variety Redpoll lyophilized juice and waste extract and their antioxidative and spasmolytic effects. The flavonol and anthocyanin contents of the samples were determined using high-performance liquid chromatography. The antispasmodic effects were assessed in in vitro conditions, and the potential impact of the samples and possible action mechanisms were revealed. The results showed the prevalence of cyanidin-3-*O*-glucoside as the dominant anthocyanin with higher content in the juice sample. Quercetin content, as the prevalent flavonol, was higher in the waste sample. The berry juice showed a greater ability for scavenging free radicals, whereas the waste better inhibited lipid peroxidation. The juice was a superior antispasmodic agent for spontaneous, KCl-, CaCl_2_-, BaCl_2_-, histamine-, and acetylcholine-induced ileal contractions. This first evaluation of the red currant variety Redpoll lyophilized juice and waste extract indicated the beneficial effects of juice as an antioxidant and spasmolytic agent. Therefore, this red currant juice sample could be designated for the prevention or treatment of gastrointestinal disorders.

## 1. Introduction

The latest research on the Global Burden of Diseases revealed that digestive disorders are the most prevalent and common type of disease affecting people worldwide [1]. This health concern can be addressed through various strategies, including prevention, screening, early detection, and treatment. The World Health Organization recommends consuming over 400 g of fruits and vegetables daily to improve health and reduce disease risks among other prevention measures [2]. Among fruits, berries and their compounds have been the subject of extensive research by scientists around the world. In addition to examining the composition, these research works also examine various biological activities, such as antioxidant, antimicrobial, anti-inflammatory, and anti-proliferative, as well as their effects on blood pressure and intestinal contractility. Contractility or motility disorders are a large group of disorders that affect the gastrointestinal tract (GIT) from the esophagus to the rectum and pelvic floor [3]. The most common symptoms of these disorders are diarrhea, abdominal pain, dyspepsia, constipation, and bloating [4]. Based on literature data, it can be seen that berries have spasmolytic properties on the small intestine in different experimental animals, but there are no data on the red currant effect on this particular tissue.

Berries are highly appreciated for their taste, high nutritional value, and health-promoting properties. Apart from other berries, currants (Ribes sp.) emerge with their sour taste and distinct polyphenol profile: anthocyanins (ACs), tannins, flavonols, and flavanols [5,6,7]. The *Ribes* L. genus consists of three subgenera: black currants (*Coreosma*), red currants (*Ribesia*), and gooseberries (*Grossularia*) [8]. Fruits of different *Ribes* species are consumed fresh or processed into syrup, juice, jams, and extracts for food supplements [9].

Red currants (RCs) are berries with a long tradition of use. RCs are rich sources of primary metabolites, such as sugars and organic acids responsible for the fruit taste. The most abundant group of secondary metabolites are polyphenols, with three most present classes in RCs: flavonoids, tannins, and phenolic acids [10]. Among flavonoids, the most dominant are ACs (cyanidin and delphidin-3-*O*-glucoside), flavonols (quercetin, kaempferol), and flavanols (catechin, epicatechin) [11,12,13,14].

Numerous health benefits of RCs may derive from their phenolic components. Epidemiological studies suggest that a polyphenol-rich diet can affect the prevention of cardiovascular diseases [15,16,17], as well as the prevention of lung cancer [18]. Several studies have found that RCs or their constituents have different biological properties, such as anti-inflammatory, anti-proliferative, antioxidant, antimicrobial, and antidiabetic [19,20,21,22].

Cyanidin-3-*O*-glucoside (C3G) is the anthocyanin that is most dispersed in various fruits. It is strongly associated with antioxidative, antiapoptotic, hepatoprotective, antimicrobial, neuroprotective, anti-inflammatory, and many more activities of the fruits [23,24,25,26,27,28].

It has been determined that the flavonols myricetin, quercetin, and kaempferol have a relaxant effect on rat and guinea ileum, both as isolated compounds or obtained from various plant sources. Kaempferol exhibits anti-inflammatory properties and has been used to cure many acute and chronic inflammatory diseases, such as colitis and post-menopausal bone loss [29]. Several papers have reported the positive effects of dietary kaempferol in reducing the risk of chronic diseases, such as cancer, obesity, and diabetes [30].

Quercetin has numerous pharmacological effects. It reduces blood pressure in both experimental animals and humans, acting as a vasodilator on human arteries in vivo and in vitro when given in an acute dose to humans with normal blood pressure and lipid status [31]. Macander and Capasso have shown that quercetin causes the relaxation of the smooth muscle [32,33]. However, there is no scientific evidence regarding the red currant to support these claims.

Therefore, bearing in mind that RCs are rich in flavonoids, the aim of this paper was to determine the content of some secondary metabolites (total phenols and tannins, ACs, and flavonol content) and the antioxidative activity of lyophilized fruit juice (Redpoll lyophilized juice—RPLJ) and waste extract (Redpoll lyophilized waste extract—RPLWE) obtained from the red currant (*Ribes rubrum* L.) variety Redpoll. The paper will also investigate the effects of RPLJ and RPLWE on the motility and contractility of rat intestinal smooth muscle in vitro, as well as the dominant anthocyanin standard.

## 2. Results

### 2.1. Chemical Content (Total Phenols, Total Tannins, Ascorbic Acid, Anthocyanin, and Flavonol Content) of RPLJ and RPLWE

The amounts of total polyphenols, tannins, and HPLC quantification of ACs, ascorbic acid (AsA), and flavonol content in the RPLJ and RPLWE are shown in Table 1. The results indicate that the RPLJ and RPLWE are rich sources of polyphenolic compounds, tannins, and AsA. A higher content of AsA was determined in RPLJ (230 mg/100 g). HPLC analysis showed that C3G was recognized as the most dominant anthocyanin in RPLJ. The content of flavonols (quercetin, kaempferol) and p-coumaric acid was higher in the RPLWE than in the RPLJ.

### 2.2. Antioxidant Activity

The results of antioxidant activity tests are presented in Table 2. The evaluation of antioxidant potency was determined in vitro using two methods. The DPPH assay examines the capacity of the extract to capture free radicals via a hydrogen atom donating mechanism. In our study, the RPLJ (0.98 ± 0.04 mg/mL) showed better antioxidant activity than the RPLWE (1.58 ± 0.20 mg/mL). The values of positive controls, i.e., BHT, BHA, AsA, and Trolox, were IC50 (μg/mL) = 23.83 ± 2.07, 2.43 ± 0.09, 6.14 ± 0.64, and 4.74 ± 0.33, respectively. The β-carotene bleaching model system determines the extract’s ability to inhibit lipid peroxidation. The best result for the β-carotene bleaching model system was achieved by the RPLWE (0.73 ± 0.07 mg/mL). The antioxidant standards used in this study, i.e., BHT, BHA, AsA, and Trolox, showed better activities in comparison to RPLJ and RPLWE (IC50 (μg/mL) = 0.02 ± 0.00, 0.03 ± 0.01, 1.68 ± 0.11, and 22.96 ± 1.52, respectively).

### 2.3. Spasmolytic Activity

#### 2.3.1. Effects of RPLJ, RPLWE, and C3G on Spontaneous Contractions of the Isolated Rat Ileum

**Figure 1:**
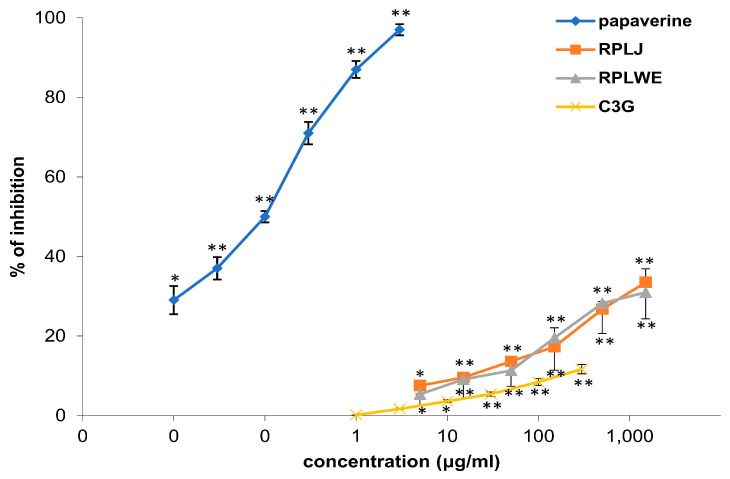
Relaxant effects of RPLJ, RPLWE, C3G, and papaverine (as a positive control) on spontaneous contractions of the isolated rat ileum. Each point represents the mean percentage values with respect to the spontaneous contractions in Tyrode solution (control) ± SD of six segments. * *p* < 0.05, ** *p* < 0.01 versus Tyrode.

Samples of RPLJ and RPJWE inhibited the spontaneous contractions of the rat ileum, and the relaxation effect itself was dose-dependent (Table 3). In our experiment, the samples showed very similar activity, with the fact that RPLJ had a slightly stronger inhibitory effect (Figure 1). The concentration of 1.5 mg/mL of RPLJ inhibited the spontaneous contraction of the ileum by 33.48 ± 8.41%, while the RPLWE at the same concentration showed an inhibitory effect of 30.97 ± 7.69%. The EC_50_ values for RPLJ and RPLWE obtained via regression analysis were 2.9 ± 0.35 mg/mL and 2.54 ± 0.20 mg/mL, respectively. C3G (5–1500 μg/mL), the main compound of the red currant, induced a spasmolytic effect by reducing spontaneous contractions of the isolated rat ileum, depending on the concentration, with an EC_50_ value of 7.17 ± 0.09 mg/mL. Papaverin (0.05–1.5 μg/mL), used as a positive control, relaxed the rat ileum in a concentration-dependent manner (EC_50_ = 7.1 ± 0.1 μg/mL). There was no significance among the EC_50_ values of the RPLJ and RPLWE (*p* < 0.05).

#### 2.3.2. Effects of RPLJ and RPLWE on KCl-Induced Contraction of Rat Ileum

**Figure 2:**
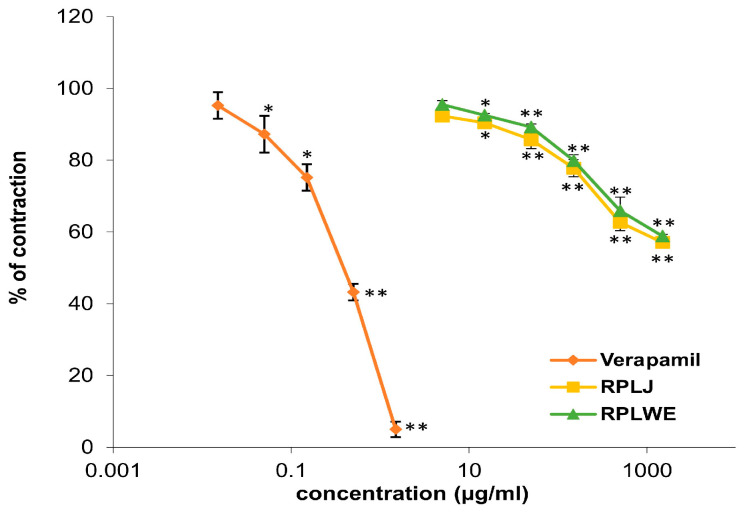
Relaxant effects of RPLJ, RPLWE, and verapamil (as a positive control) on the KCl-induced contractions of the isolated rat ileum. Each point represents the mean values in percent of maximal response ± SD of six segments. Statistical analysis was conducted using Student’s *t*-test, with * *p* < 0.05, ** *p* < 0.01 indicating significance compared to the control.

Various concentrations of RPLJ and RPLWE (0.005–1.5 mg/mL) showed relaxant effects on the rat ileum precontracted with 80 mM KCl solution (Figure 2). RPLJ at a concentration of 0.3 mg/mL relaxed the contractions of the rat ileum by 42.9 ± 0.75%, while RPLWE at the same concentration showed lower relaxation values (41.13 ± 0.65%). The effective doses that inhibited 50% of KCl-induced ileum contraction (EC_50_) were 1.62 ± 0.02 mM for RPLJ and 1.7 ± 0.025 mM for RPLWE. Verapamil, used as a control, reduced the contractions even further to 5% with a maximum concentration of 0.0015 mg/mL. The EC_50_ value was 6.3 × 10^−4^ ± 0.5 × 10^−4^ mM. There was no significance among the EC_50_ values of the RPLJ and RPLWE (*p* > 0.05).

#### 2.3.3. Effects of RPLJ and RPLWE on Histamine-Induced Ileum Contraction

**Figure 3:**
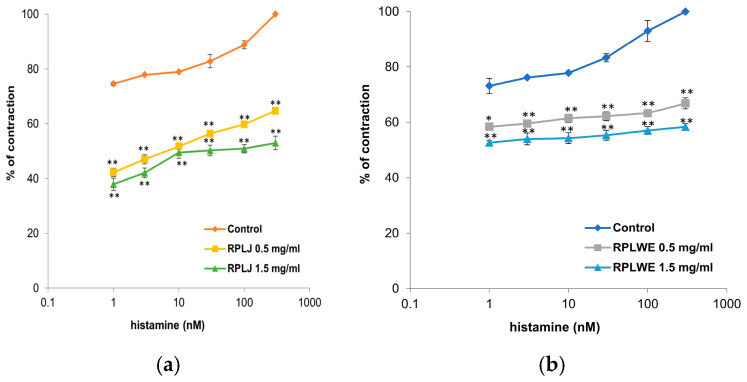
Dose-dependent relaxation effects of RPLJ and RPLWE on the histamine-induced contractions of the isolated rat ileum. The graphs show (**a**) the effects of the RPLJ on histamine dose–response curves (0.5 mg/mL and 1.5 mg/mL), (**b**) the effects of the RPLWE on histamine dose–response curves (0.5 mg/mL and 1.5 mg/mL). Each point represents the mean values in percent of maximal response ± SD of six segments. Statistical analysis was conducted using Student’s *t*-test, with * *p* < 0.05, ** *p* < 0.01 indicating significance compared to control.

Histamine (1–300 nM) was used to stimulate the contraction of isolated rat ileum. RPLJ significantly reduced contractions caused by histamine depending on the concentration, while RPLWE did not show as significant a reduction in contractions as RPLJ at the same concentrations (Figure 3). RPLJ applied at a concentration of 0.5 mg/mL relaxed the isolated rat ileum by 32.37 ± 1.45% in contractions induced by 1 nM of histamine and by 35.35 ± 2.8% in contractions induced by 300 nM of histamine. RPLJ at a concentration of 1.5 mg/mL inhibited contractility by 36.68 ± 3.1% and 47.04 ± 4.4% for contractions induced by 1 nM and 300 nM of histamine, respectively. On the other hand, RPLWE applied at a concentration of 0.5 mg/mL relaxed the isolated rat ileum by 14.77 ± 0.3% in contractions induced by 1 nM of histamine and by 33.1 ± 1.61% in contractions induced by 300 nM of histamine, while RPLWE at a concentration of 1.5 mg/mL induced relaxation of 20.51 ± 0.8% and 41.64 ± 3.9% for contractions induced by 1 nM and 300 nM of histamine, respectively. There was a statistically significant difference in spasmolytic activity between juice at lower and higher concentrations, as well as waste at lower and higher concentrations (*p* < 0.05, *p* < 0.01).

#### 2.3.4. Effects of RPLJ and RPLWE on Acetylcholine (ACh)-Induced Contraction of Rat Ileum

**Figure 4:**
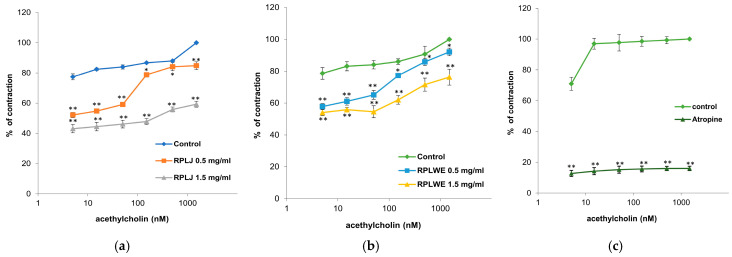
Relaxant effects of RPLJ and RPLWE on the ACh-induced contraction of rat ileum. The graphs show (**a**) the values of control, ACh + RPLJ (0.5 mg/mL), ACh + RPLJ (1.5 mg/mL), (**b**) the values of control, ACh + RPLWE (0.5 mg/mL), ACh + RPLWE (1.5 mg/mL), (**c**) the values of control and atropine (used as a positive control). Each point represents the mean values in percent of maximal response ± SD of six segments. Statistical analysis was conducted using Student’s *t*-test, with * *p* < 0.05 and ** *p* < 0.01 indicating significance compared to the control.

In this experimental series, the relaxing effect of RPLJ and RPLWE on ACh-induced contraction of rat ileum (5–1500 nM) was also tested. Both samples (at concentrations of 0.5 mg/mL and 1.5 mg/mL) inhibited the induced ileum contractions in a concentration-dependent manner (Figure 4). The EC_50_ value of ACh was 0.001 ± 0.0001 nM. RPLJ and RPLWE (0.5 mg/mL) increased the EC_50_ to 5.23 ± 0.05 nM and 2.35 ± 0.03 nM, respectively. When the increased concentrations of RPLJ and RPLWE (1.5 mg/mL) were applied, the effective doses that inhibited 50% of small intestine ACh-induced contractions were 104.39 ± 1.34 nM and 4.34 ± 0.04 nM, respectively. There was a statistically significant difference in spasmolytic activity between juice at lower and higher concentrations, as well as waste at lower and higher concentrations (*p* < 0.05).

#### 2.3.5. Effect of RPLJ and RPLWE on BaCl_2_-Induced Contraction of Rat Ileum

**Figure 5:**
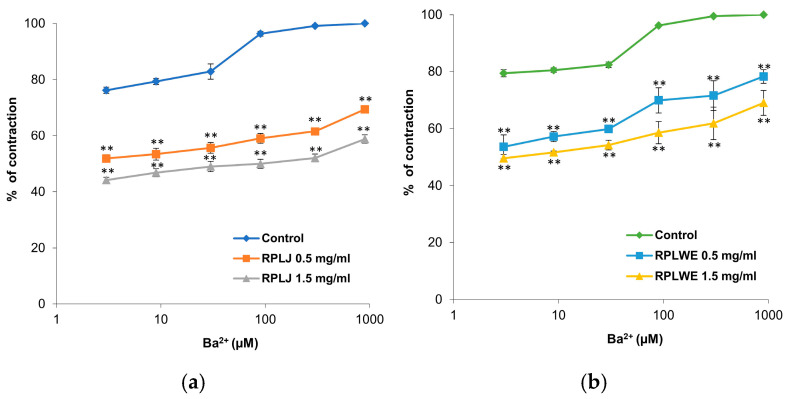
Dose-dependent relaxing effects of the RPLJ and RPLWE on the BaCl_2_-induced contraction of rat ileum. The graphs show (**a**) the effects of the RPLJ on Ba^2+^ dose–response curves (0.5 mg/mL and 1.5 mg/mL), (**b**) the effects of the RPLWE on Ba^2+^ dose–response curves (0.5 mg/mL and 1.5 mg/mL). The data point represents the mean value of the response, accompanied by the SD of six segments. Statistical analysis was conducted using Student’s *t*-test, with ** *p* < 0.01 indicating significance compared to the control.

The effects of RPLJ and RPLWE on BaCl_2_-induced ileum contractions (3–900 μM) are shown in Figure 5. The samples reduced BaCl_2_-stimulated contractions of isolated rat ileum in a concentration-dependent manner. The EC_50_ value of BaCl_2_ was 0.015 ± 0.18 μM. In the presence of RPLJ and RPLWE (0.5 mg/mL), the EC_50_ values increased to 2.76 ± 1.5 μM for juice and 1.69 ± 1.2 μM for waste. On the other hand, when applied at a concentration of 1.5 mg/mL, RPLJ and RPLWE changed the EC_50_ values to 49.62 ± 9.1 μM and 5.36 ± 3.1 μM, respectively. There was a statistically significant difference in spasmolytic activity between juice at lower and higher concentrations, as well as waste at lower and higher concentrations (*p* < 0.05).

#### 2.3.6. Effects of RPLJ and RPLWE on CaCl_2_-Induced Contraction of Rat Ileum

**Figure 6:**
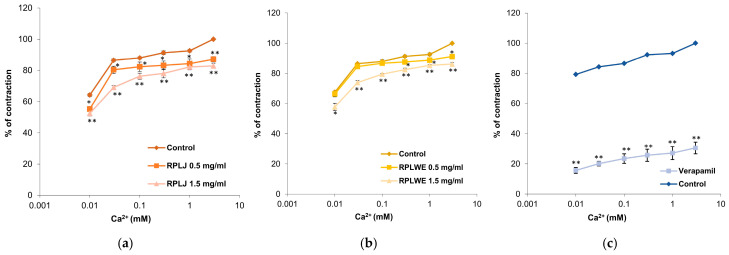
Relaxant effects of RPLJ, RPLWE, and verapamil on CaCl_2_-induced contraction of rat ileum. The graphs show (**a**) the effects of the RPLJ on Ca^2+^ dose–response curves (0.5 mg/mL and 1.5 mg/mL), (**b**) the effects of the RPLWE on Ca^2+^ dose–response curves (0.5 mg/mL and 1.5 mg/mL), (**c**) the values of control and verapamil (used as a positive control). Each point represents the mean values in percent of maximal response ± SD of six segments. Statistical analysis was conducted using Student’s *t*-test, with * *p* < 0.05 and ** *p* < 0.01 indicating significance compared to the control.

Inhibitions of ileum contractions caused by the action of calcium ions (0.01–3 mM) were examined in the presence of RPLJ and RPLWE (Figure 6). RPLJ at a concentration of 0.5 mg/mL reduced the contraction of the ileum and modified the EC_50_ value of CaCl_2_ from 1 × 10^−4^ ± 0.1 × 10^−4^ mM to 2 × 10^−4^ ± 0.1 × 10^−4^ mM, while at a concentration of 1.5 mg/mL, the EC_50_ increased to 1.3 × 10^−3^ ± 0.3 × 10^−3^ mM. RPLWE did not show relaxant effect. Its inhibition of maximal contractions at a concentration of 0.5 mg/mL was reduced to an EC_50_ value of 8 × 10^−6^ ± 0.1 × 10^−6^ mM, while the EC_50_ at a concentration of 1.5 mg/mL was 3.6 × 10^−4^ ± 0.1 × 10^−4^ mM. Verapamil, a calcium channel antagonist, as expected, was the best inhibitor of the Ca^2+^-induced contractions, reducing them to 30.54 ± 3.92% at a dose of 0.3 µM. There was a statistically significant difference in spasmolytic activity between juice at lower and higher concentrations, as well as waste at lower and higher concentrations (*p* < 0.05).

## 3. Discussion

Berries are a rich nutritional source of bioactive compounds containing ACs, phenolic acids, proanthocyanidins, ascorbic acid, and minerals [34]. The literature data have shown that considerable numbers of active substances are affected by environmental conditions (season, degree of maturity, growing conditions) [35]. The chemical analyses confirmed that our samples were rich sources of polyphenolic compounds, tannins, ACs, and AsA. The amounts of total phenolics and tannins presented in Table 1 show that RPLJ contained a higher amount of total phenolics and tannins than RPLWE. Djordjevic reported higher total phenolics in fresh juice of the same variety than those reported in our study (90.8 ± 1.8 mg GAE/100 g fresh weight) [11]. The difference in content comes from the difference in the sample, year of harvest, and place of cultivation. Plessi et al. have noticed that the amount of total phenol content depends on the solvent used in the extraction, with the lowest value obtained via water extraction [36]. Biochemical analysis showed that the RPLJ had larger amounts of ACs and AsA. The major anthocyanin was C3G, identified via the HPLC analysis. Similar results were obtained by Djordjevic [11] and Wu [37], where C3G was found to be predominant but at a lower concentration than in our study. The amount of C3G was 155.38 ± 7.34 mg/100 g in RPLJ and 34.3 ± 4.55 mg/100 g in RPJWE. In addition to C3G, C3R was also determined. The amount of C3R was also higher in RPLJ (28.34 ± 3.51 mg/100 g) than in RPJWE (8.4 ± 0.78 mg/100 g).

In this research, a higher content of AsA was determined in RPLJ (230 mg/100 g). The literature data have shown concentrations of AsA in several varieties of red currant, although the samples were fresh fruits, so the results cannot be fully compared. Certain variability seems to exist among the levels of ascorbic acid between this study and previous studies [11,12,21,38,39,40]. This difference could be explained by the fact that the varieties tested were different. In addition, the analytical techniques and the working conditions were different. Pantelidis determined the level of AsA in two RC varieties: London Market and Rovada. The contents of AsA were 35.6 ± 2.2 (mg 100 g^−1^ fw) for the London Market variety and 40.0 ± 2.3 (mg 100 g^−1^ fw) for the Rovada variety [39]. Rotundo and others determined the content of AsA to be 65.0 mg/100 g FW for *R. rubrum* [10]. Djordjevic et al. analyzed the content of AsA of eleven red currant cultivars (Junifer, Jonkheer van Tets, Rolan, Stanza, Rondom, Mirana, Rovada, London Market, Makosta, Redpoll, and Slovakia). The Junifer variety was the richest (71.6 mg/100 g), whereas the London Market variety was the poorest (50.5 mg/100 g). However, the samples studied by Djordjevic were from different regions and years [11].

The radical scavenging activity toward the stable DPPH radical was applied in the study of Djordjevic et al., where RC varieties were tested against the DPPH radical, and the results showed that juice of the Redpoll variety showed the strongest DPPH radical scavenging activity, with an IC50 value of 1.9 mg/mL. The weakest scavenging activity toward the DPPH radical was noted in the Slovakia variety, with an IC50 value of 12.3 mg/mL [11]. Our results showed stronger antioxidant activity compared to the same variety in the study by Djordjevic. Benvenuti et al. determined the DPPH in three varieties of R. rubrum (cv. Rotet, Red Lake, and Rosetta). The DPPH scavenging activity was higher in the Rotet variety, with a value of 4.3 ± 0.6 mg, and the Red Lake variety had the lowest activity, with a value of 5.9 ± 0.8 mg [12]. The difference in the obtained results can be explained by the fact that the cultivars tested were not the same and that environmental factors strongly influence each variety. This conclusion was confirmed in the research of Mikulic-Petkovsek and others [41] and Savikin and others [42].

On the other hand, the best inhibition of lipid peroxidation in the *β*-carotene/linoleic acid emulsion was observed in RPLWE. There have been no literature data on the topic of the *β*-carotene bleaching method in RCs.

Numerous studies suggest that food rich in flavonols can alleviate cardiovascular disease, diabetes, cancer, and viral and bacterial infections [43,44,45,46,47]. Gastrointestinal problems are chronic symptoms that indicate the digestive tract’s dysfunction, especially in the small intestine. Flavonols (quercetin and kaempferol) are known for their potent antioxidant properties and beneficial effects on GIT problems [48,49]. A study by Carlo confirmed that quercetin reduced intestinal transit and motility in mice [50]. Other studies showed that quercetin had a calcium antagonist effect, as well as a relaxant effect on smooth muscle contraction. Quercetin can induce relaxation of human gastric smooth muscle directly through the K^+^ATP channels and a relaxant effect on isolated smooth muscle contractions [51,52]. Kaempferol is also known for its activity in smooth muscles. There are data that demonstrate that it can inhibit calcium-mediated signals and the expression of target genes involved in smooth muscle contraction [53]. Santos-Fagundes showed that quercetin had an inhibitory effect on the spontaneous contractions of rabbit duodenum [54]. Miladinovic et al. confirmed the spasmolytic effect of quercetin on the intestinal smooth muscle of rats [55]. Previous studies have mostly addressed the chemical profile of RC, but the literature data on motility and contractility of the rat ileum smooth muscle in vitro induced by the RPLJ and RPLWE have not been investigated so far. Our results demonstrated that RPLJ induced better inhibition of spontaneous contractions of the rat ileum than RPLWE. We can attribute such a result to the fact that there may be a synergistic effect of phenolic compounds, which can lead to a decrease in the contractions’ tone in the rat ileum musculature.

Our samples showed relaxant effects on the small intestine of rats precontracted with KCl solution. High concentration of potassium ions causes tonic contractions of smooth muscles due to membrane depolarization, the opening of L-type voltage-dependent calcium channels, and entry of Ca^2+^ into the cell [56]. It depolarizes the smooth muscle preparations and is known to produce myo-contractions through the opening of voltage-dependent Ca^2+^ channels, thus allowing an influx of extracellular Ca^2+^, resulting in a contractile effect [57,58]. As RPLJ and RPLWE inhibit KCl-induced contractions, they can be considered calcium channel blockers. This mechanism of spasmolytic activity, which implies the opening of potassium channels and the blocking of calcium channels, is the most common one in plant extract actions [59]. In our study, RPLJ had better relaxant effects on the rat ileum.

Histamine application on the smooth muscles of the gastrointestinal tract can lead to a variation in responsiveness and sensitivity of their different regions, membrane depolarization, and increased excitability [60]. In our study, RPLJ inhibited histamine-induced contractions, while RPLWE did not result in a relaxant effect.

Acetylcholine is a neurotransmitter, which has numerous physiological functions and, most importantly, regulates intestinal peristalsis. In GIT, ACh stimulates the nervus vagus and increases the tone and amplitude of stomach and intestine contractions [61]. Acetylcholine leads to contractions of the rat ileum via muscarinic receptors with two mechanisms. The first one works by activating non-selective cation channels in the cell membrane, which leads to membrane depolarization, the opening of voltage-dependent Ca^2+^ channels, and entry of Ca^2+^ ions into the cell. The second one works by releasing intracellular Ca^2+^ ions [62]. In our study, RPLJ and RPLWE inhibited contractions induced by ACh. RPLJ had better relaxant effects on the rat ileum than RPLWE.

Contractions of the rat ileum caused by the addition of CaCl_2_ solution were alleviated by RPLJ, while RPLWE did not lead to a relaxant effect. Barium is a non-specific blocker of K^+^ channels, and it causes membrane depolarization and contraction of the ileum smooth muscles [63].

As stated above, there are no published studies on the spasmolytic effect of red currant. Some authors have studied the effect of plant extracts on the rat ileum. Miladinovic demonstrated the effect of black currant juices on the spontaneous motility and contractility of rat intestinal smooth muscle [55,64].

Our results demonstrated that the RPLJ and RPLWE relaxed spontaneous contractions in the isolated rat ileum, which was induced by different agents. The spasmolytic effects could be explained by the activity of the main compounds in RCs.

## 4. Materials and Methods

### 4.1. Plant Material and Sample Preparation

Red currant of the Redpoll variety was collected from Radmilovac (Faculty of Agriculture University of Belgrade). Berries were harvested in 2020, from June to July. After collection, berries were pressed in a special press (Bucher Vaslin, Chalonnes-sur-Loire, France), and juice was immediately frozen and stored at a temperature of −18 °C. The waste (residue remaining after straining the juice) was dried at 40 °C for 48 h in a laboratory dryer (Instrumentaria ST 01/02, Zagreb, Croatia) and subsequently ground in a mill (UMČ-20, Biljotehnika, Pančevo, Serbia). The shredded waste was sifted using a sieve, according to the regulations of the Yugoslavian Pharmacopoeia (Ph. Yug. V, 2000 [65]), to obtain a fraction of 0.75–2 mm. The maceration method was used to extract the plant material (60% ethanol was used with a solid-to-solvent ratio of 1:20). The waste was extracted for 60 min at room temperature on a laboratory shaker (Unimax 1010, Heidolph, Schwabach, Germany) with 170 rpm rotation [66]. After the extraction process, the obtained liquid extracts were separated from the residue by filtering; subsequently, ethanol was removed using a rotary evaporator (Büchi CH, Flawil, Switzerland). The remaining waste extract was stored in closed containers at −18 °C. The sample thus obtained was used for lyophilization.

The juice and waste extracts were frozen at −80 °C and lyophilized at −60 °C (at a pressure of 0.011 mbar) for twenty-four hours and at −60 °C (at a pressure of 0.0012 mbar) for an additional hour to remove capillary water residues (Beta 1–8 Freeze Drier, Martin Christ, GmbH, Osteroide am Harz, Germany). The obtained lyophilized samples were labeled Redpoll lyophilized juice (RPLJ) and Redpoll lyophilized waste extract (RPLWE).

### 4.2. Determination of Total Polyphenols

The determination of total polyphenols was performed using the Folin–Ciocalteu (F-C) colorimetric method, according to Makkar [67]. Spectrophotometric determination of the sample and standard is valid with absorbance at 725 nm. The results were expressed as gallic acid equivalents (GAE) (mg GAE/g juice or extract).

### 4.3. Determination of Total Tannins

The content of total tannins was determined using the method outlined by Makkar [67]. The determination of total tannins was performed with the F-C reagent using insoluble polyvinylpolypyrrolidone (PVPP), which binds tannins in solution. The difference between total and non-tannic polyphenols represents the tannin content. The results were expressed as gallic acid equivalents (mg GAE/g juice or extract).

### 4.4. Determination of ACs

The measurement of ACs was performed as described by Cacace and Mazza [68], with slight modification. Lyophilized juice/extract (10 mg) was dissolved in 1 mL of deionized water acidified with sulfuric acid to pH 2.5. The sample was treated in an ultrasonic bath for 10 min and centrifuged at 4000 rpm at room temperature. The supernatant was taken for HPLC analysis after filtering through a Millipore membrane filter (0.45 µm).

#### Chromatographic Conditions

The analysis was performed with Agilent 1200 (Agilent Technologies, Palo Alto, CA, USA) equipped with a diode array detector (DAD), an automatic sampler, and a control system. The separation of components was performed using Merck Purospher STAR RP-18e analytical column (150 × 4.6 mm i.d., 5 μm particle size). Trifluoroacetic acid 0.1% was used as mobile phase A, and acetonitrile was used as mobile phase B. The separation was conducted under isocratic conditions, with a flow rate of 1 mL/min at 25 °C. A volume of 20 µL of the sample was injected, and AC detection was performed at 520 nm. The AC content was calculated using a calibration curve, which was constructed based on standard solutions of 2 ACs (C3R and C3G). Standard solutions were injected and detected in triplicate at 5 increasing concentrations (0.2, 1, 5, 20, and 50 µg/mL).

### 4.5. Ascorbic Acid Determination (AsA)

AsA and dehydroascorbic acid (DAsA) were determined using the HPLC method according to Brubacher [69] for the preparation sample and Ohta and Harada [70] for DAsA reduction. An amount of 10 mg of lyophilized juice/extract was dissolved in 1 mL of 4.5% metaphosphoric acid. The sample was dissolved in ultrasonic bath for 10 min and centrifuged for 15 min at 10,000 rpm. The supernatant obtained with this method was used to determine AsA. DAsA was determined when 1 mL of the reducing agent (50 μM 1,4-dithiothreitol—DTT) was added to 1 mL of the previously prepared sample. After a 10 min reaction at room temperature, this solution was filtered, and 20 μL was injected into HPLC. The results were expressed in µg/100 mg of RPLJ and RPLWE as a total amount of ascorbic acid (TAsA = AsA + DAsA).

#### Chromatographic Conditions

The same system was utilized both for the separation and quantification of AC and the determination of AsA. The mobile phase used 40 mM phosphate buffer and methanol at a ratio of 92:8 under isocratic conditions. The flow rate was 0.8 mL/min at room temperature, with absorbance at 244 nm. The standard solutions of AsA (1, 10, 50, 100, 150, and 200 μg/mL) were injected in triplicate for each concentration to obtain a calibration curve.

### 4.6. Determination of Flavonol Content

The sample preparation involved previous hydrolysis of flavonols. The extract (10 mg) was dissolved in 1 mL of methanol and hydrochloric acid mixture (1:1, *V*/*V*). The dissolution was carried out in an ultrasonic bath for 10 min and then in a water bath at a temperature of 90 °C for 15 min. After hydrolysis, the samples were centrifuged for 20 min (4 °C, 10,000 rpm). The supernatants were taken for HPLC analysis after filtering [55].

#### Chromatographic Conditions

The same chromatographic system was used for separating and quantifying the flavonols, as well as for determining the ACs and AsA. Trifluoroacetic acid 0.1% was used as mobile phase A, and acetonitrile was used as mobile phase B. The separation of flavonoids was carried out at the following gradient: 85% (A) from 0 to 20 min; 65% (A) from 20 to 24 min; 50% (A) from 24 to 29 min; then 10% (A) to 31 min; and 15% for the next 2 min. A volume of 10 µL of the sample was injected, with a flow rate of 0.7 mL/min. The column temperature was maintained at 30 °C, and detection was performed at 360 nm. Flavonol contents were determined from the calibration curves, which were constructed based on the flavonol standards: quercetin and kaempferol. The results were expressed in µg of flavonols/100 mg of the extract.

### 4.7. 2,2-Diphenyl-1-picrylhydrazyl (DPPH) Radical Scavenging Activity

The scavenging of DPPH radicals was investigated according to the Konić-Ristić method, with minor changes [39]. In total, 3 mg of lyophilized juice and waste was dissolved in 1 mL of distilled water and ethanol, respectively, and sonicated for 5 min in ultrasonic bath. The solution was centrifuged for 10 min at 2500 rpm, and the supernatant was used for analysis. This solution was diluted to seven decreasing concentrations. Each of these diluted samples (40 μL) was mixed with methanol (120 μL) and the DPPH solution (40 μL; 0.05 mM) in a microtiter plate and left in the dark for 30 min, when the reaction reached a plateau. Absorbance of the samples was measured on an enzyme-linked immunosorbent assay (ELISA) reader (Multiskan Ascent Thermolabsystems Elisa No 354, Thermo Fisher Scientific, Vantaa, Finland) at a wavelength of 540 nm. Inhibition of the free DPPH radical (%) was calculated according to the following equation:

% inhibition = (Ac − As/Ac − AB) × 100

where AB is the absorbance of the blank test (the solvent); Ac is the absorbance of the control (consisting of the solvent and DPPH); and As is the absorbance of the analyzed sample. The solvent was used as a blank (methanol). Butylhydroxy toluene (BHT), butylhydroxy anisole (BHA), Trolox (6-hydroxy-2,5,7,8-tetramethylchroman-2-carboxylic acid), and ascorbic acid were used as a positive control. The DPPH radical inhibition results were expressed as IC50, and this value represents the sample concentration required to “capture” or inhibit 50% of the free DPPH of radicals, and it is calculated from the line equation, which represents the ratios of % inhibition and sample concentrations.

### 4.8. β-Carotene Bleaching Method

The method was developed based on the spectrophotometric measurements of Koleva [71]. The method determines the antioxidant capacity of the sample by measuring its ability to prevent oxidative loss of β-carotene in the β-carotene/linoleic acid emulsion. The emulsion is prepared in the following manner: 2 mg of crystalline β-carotene is dissolved in 10 mL of chloroform. Amounts of 25 µL of linoleic acid and 180 mg of Tween 20 are added to 1 ml of this solution. After complete evaporation of chloroform with a vacuum evaporator at 40 °C, 50 mL of oxygenated water is added, and the emulsion thus obtained is shaken until it becomes clear. Aliquots of the emulsion (0.16 mL) are pipetted into the wells of the microtiter plates, which contain the previously added dilution series of juices or extracts (0.04 mL). The microtiter plates are then stirred on a mixer for microtiter plates. After mixing, the initial absorbance (A0) was read on an ELISA reader at a wavelength of 450 nm. The plates were incubated for 2 h at 55 °C, after which absorbance (A2h) was read again. The antioxidative activity was calculated according to the following formula:

% inhibition = (A2h/A0) × 100

BHA, BHT, Trolox, and ascorbic acid were used as positive controls. The results were expressed as the concentration of the sample that inhibited the loss of 50% of *β*-carotene (IC50), and this was calculated from the concentration/% inhibition curve.

### 4.9. Spasmolytic Activity

#### 4.9.1. Experimental Animals

To define a spasmolytic activity, we used male albino laboratory rats of the Wistar strain (bodyweight 200–250 g, aged 10–12 weeks, bred in a vivarium in the Faculty of Medicine, University of Niš). One week before the experiment, the animals were separated and kept in stainless-steel wire cages under standard laboratory conditions with a room temperature maintained between 20 and 24 °C, with a 12 h daily regimen. The animals had access to food and water at all times, and food was withdrawn 24 h before the experiment.

The experimental procedures were performed in accordance with the European Directive 2010/63/EU on experiments involving animals, with approval granted by the Veterinary Administration of the Ministry of Agriculture and Environmental Protection (decision number 323-07-09101/2020-05/2).

#### 4.9.2. Experimental Design

At the beginning of the experiment, rats were placed in a closed chamber and exposed to ether vapors. After anesthetization, the chest of the rats was opened, and the aorta was cut; then, parts of the ileum were isolated. Parts of the ileum prepared in this way were placed in a bath containing 20 mL of saline solution for the isolated intestine (Tyrod’s solution) at 37 °C, with constant introduction of the oxygen and carbon dioxide mixture (95% and 5%). Half an hour before the start of the experiment, the ileum was stabilized in the bathroom [72]. Changes in contractility of the small intestine were registered using a transducer (Transducer-TSZ-04-E, Experimetria doo, Budapest, Hungary), and the obtained data were analyzed with the help of SPEL Advanced ISOSYS Data software Acquisition System (Experimetria Ltd., Budapest, Hungary).

The influence of RPLJ and RPLWE, dissolved in Tyrode solution, on the concentration required was monitored on spontaneous and induced (stimulated) contractions of the isolated ileum. The contractions were stimulated using acetylcholine (ACh), potassium chloride (KCl), barium chloride (BaCl_2_), calcium chloride (CaCl_2_), and histamine.

#### 4.9.3. Examination of the Effect of RPLJ, RPLWE, and C3G on Spontaneous Contractions of the Rat Ileum

The first experimental series consisted of analysis of the effects of RPLJ and RPLWE on spontaneous contractions of the isolated ileum. The extract solutions (0.005, 0.015, 0.05, 0.15, 0.5, and 1.5 mg/mL) and C3G (5–1500 μg/mL) were added in cumulative doses, whereby a dose-dependent response was read from the curve. The extracts were added after the adaptation period. The results were calculated as the difference between the curve area before and after the addition of samples. For each concentration of the extract, the spasmolytic effect was expressed as a percentage of the initial spontaneous activity of the isolated intestine without the presence of the examined extract (55). Papaverin was used as a positive control at concentrations of 0.005–1.5 μg/mL.

#### 4.9.4. Examination of the Effect of RPLJ and RPLWE on KCl-Induced Contraction of the Rat Ileum

Ileum contractions were induced by adding KCl solution (80 mM) after the adaptation period. The resulting tonic contractions of the ileum induced by KCl were inhibited by cumulative additions of the juice/waste at concentrations from 0.005 to 1.5 mg/mL at intervals of 15 min. The spasmolytic effect of the extracts was calculated based on the differences between the areas under the curve and the basal lines as a percentage of inhibition of the KCl effect [55].

#### 4.9.5. Examination of the Effect of RPLJ and RPLWE on CaCl_2_-Induced Contraction of the Rat Ileum

For this experiment, the adaptation period of the ileum preparation was achieved in an ion-free calcium solution. After the establishment of stable spontaneous contractions, increasing concentrations of CaCl_2_ (0.01, 0.03, 0.1, 0.3, 1, and 3 mM) were added to the water bath, and increasing contractions were recorded. Based on the obtained results, a control curve was constructed. The ileum preparation was then flushed again until stable spontaneous contractions were induced. Afterward, a 0.5 mg/mL extract solution was added to the water bath, and after five minutes, the same increasing concentration of CaCl_2_ was added. The procedure was repeated with the extract solution concentration of 1.5 mg/mL. Relaxant effects of the tested extracts were shown through a series of curves showing the contractile effect of CaCl_2_ (%), which was achieved in the presence of the tested samples and was compared with the effects of the control series, in which it acted only with CaCl_2_, i.e., in the absence of extract. Verapamil was administrated as a standard at a dose of 0.3 µM [55].

#### 4.9.6. Examination of the Effect of RPLJ and RPLWE on BaCl_2_-Induced Contraction of the Rat Ileum

Increasing concentrations of the BaCl_2_ solution (3, 9, 30, 90, 300, and 900 μM) were added into the water bath after the adaptation period to induce contractions of the ileum preparation. The experiment examined the responses of RPLJ and RPLWE (0.5–1.5 mg/mL) to contractions of the isolated rat ileum induced by BaCl_2_. The spasmolytic effect of the tested samples was presented through a series of curves showing the contractile effect of BaCl_2_ (%) achieved in the presence and absence of the tested samples [72].

#### 4.9.7. Examination of the Effect of RPLJ and RPLWE on ACh-Induced Contraction of the Rat Ileum

In this series of experiments, ileum contractions were stimulated by the cumulative addition of increasing concentrations of ACh (5, 15, 50, 150, 500, and 1500 nM) after an adaptation period. The experiments involved the responses of RPLJ and RPLWE (0.5–1.5 mg/mL) to contractions induced by ACh on the isolated rat ileum. After the addition of ACh, we constructed a calibration curve before and after the addition of RC juice and waste extracts. The effect of RPLJ and RPLWE on ACh-induced contraction of the ileum was expressed as a percentage of the control response mediated by the agonists [55].

#### 4.9.8. Examination of the Effect of RPLJ and RPLWE on Histamine-InducedContraction of the Rat Ileum

Increasing concentrations of histamine solution were added to induce ileum contractions (1, 3, 10, 30, 100, and 300 nM) into the water bath. The dependence of the obtained contractions (%) in relation to the concentration of histamine solution is represented by the control curve. Afterward, preparations of the ileum were flushed with Tyrode’s solution until spontaneous contractions were re-established. The tested RPLJ/RPLWE (1.5 mg/mL) was then added to the water bath, and after 5 min, repeat series with the same concentrations of histamine solution (1–300 nM) were added. For the purposes of assessing the spasmolytic effect of the RPLJ and RPLWE, the curves of the contractile effect of histamine (%) were constructed in the presence of 1.5 mg/mL RPLJ or RPLWE and their absence [73].

### 4.10. Statistical Analysis

The results for TP, TT, ACs, flavonols, AsA, and AO activity were expressed as mean value ± standard deviation (SD), since all measurements were conducted in triplicate. The EC_50_ values (the concentration resulting in 50% of the maximum response) were obtained via regression analysis. Significant statistical differences among the EC_50_ values of RPLJ and RPLWE were obtained using Student’s *t*-test or one-way ANOVA along with Duncan’s post hoc test (*p* < 0.05 or *p* < 0.01). The obtained results were analyzed using the SPSS statistical software package (version 25.0; Chicago, IL, USA).

## 5. Conclusions

This research showed, for the first time, that the Redpoll lyophilized juice and waste exhibited good spasmolytic activities with regard to the rat ileum. This study also proved that the RPLJ and RPLWE exhibited strong antioxidant effects. The juice was richer in polyphenols, flavonols, and ACs and showed better antioxidant activity through the free radical scavenging mechanism and stronger inhibitory effect on spontaneous and ACh-, KCl-, CaCl_2_-, BaCl_2_-, and histamine-induced contractions. This study also encourages and supports the usage of Redpoll lyophilized juice and waste in the treatment of oxidative stress and gastrointestinal disorders.

## Figures and Tables

**Table 1:** Chemical content and antioxidative activity of RPLJ and RPLWE.

Compounds	Units	RPLJ	RPLWE
TP	mg GAE/g	1.34 ± 0.07 a	1.06 ± 0.04 a
TT	mg GAE/g	0.98 ± 0.05 a	0.76 ± 0.02 a
TAsA	mg/100 g	230.00 ± 0.01 a	20.00 ± 0.01 b
C3R	mg/100 g	28.34 ± 3.51 a	8.40 ± 0.78 b
C3G	mg/100 g	155.38 ± 7.34 a	34.30 ± 4.55 b
quercetin	mg/100 g	14.00 ± 0.76 a	18.60 ± 0.97 a
kaempferol	mg/100 g	2.20 ± 0.03 a	3.50 ± 0.24 a
p-coumaric acid	mg/100 g	n.d a	82.60 ± 4.45 b

Each value represents the means of three replicate determinations ± standard deviations. TP—total phenols, TT—total tannins, TAsA—total ascorbic acid, C3R—cyanidin-3-*O*-rutinoside, C3G—cyanidin-3-*O*-glucoside, n.d. = not detected. Values with different letters in the row (a,b) are significantly different according to Student’s *t*-test at *p* < 0.05.

**Table 2:** Antioxidative activity of RPLJ and RPLWE in mg/mL estimated by 2,2-diphenyl-1-picrylhydrazyl (DPPH) radical scavenging and β-carotene/linoleic acid systems.

Sample	DPPH (mg/mL)	*β*-Carotene (mg/mL)
RPLJ	0.98 ± 0.04 a	3.93 ± 0.09 a
RPLWE	1.58 ± 0.20 a	0.73 ± 0.07 b

The results present mean values of three measurements ± standard deviations. Values with different letters in the column (a,b) are significantly different according to Student’s *t*-test at *p* < 0.05.

**Table 3:** EC_50_ values (mg/mL) of the spontaneous contractions for RPLJ, RPLWE, C3G, papaverine (positive control).

Sample	EC50
RPLJ	2.90 ± 0.35 a
RPLWE	2.54 ± 0.20 a
C3G	7.17 ± 0.09 b
papaverine	7.1 × 10^−3^ ± 0.10 × 10^−3^ c

The results represent the means of six measurements ± the standard deviation (SD). Values in the column with different letters (a,b,c) are significantly different according to ANOVA with Duncan’s post hoc test at *p* < 0.05.

## Data Availability

Data are contained within the article.
